# Utility of diffusion tensor imaging and generalized q-sampling imaging for predicting short-term clinical effect of deep brain stimulation in Parkinson’s disease

**DOI:** 10.1007/s00701-024-06096-w

**Published:** 2024-05-15

**Authors:** Sabahattin Yuzkan, Ozan Hasimoglu, Serdar Balsak, Samet Mutlu, Mehmet Karagulle, Fadime Kose, Ayca Altinkaya, Bekir Tugcu, Burak Kocak

**Affiliations:** 1 Department of Radiology, Koc University Hospital, Istanbul, Turkey; 2 Department of Neurosurgery, University of Health Sciences, Basaksehir Cam and Sakura City Hospital, Istanbul, Turkey; 3 Department of Radiology, Bezmialem Vakif University Hospital, Istanbul, Turkey; 4 Department of Radiology, University of Health Sciences, Basaksehir Cam and Sakura City Hospital, Basaksehir, Istanbul, 34480 Turkey

**Keywords:** Deep brain stimulation, Diffusion tensor imaging (DTI), Generalized q-sampling imaging (GQI), Magnetic resonance imaging (MRI), Parkinson’s disease

## Abstract

**Purpose:**

To assess whether diffusion tensor imaging (DTI) and generalized q-sampling imaging (GQI) metrics could preoperatively predict the clinical outcome of deep brain stimulation (DBS) in patients with Parkinson’s disease (PD).

**Methods:**

In this single-center retrospective study, from September 2021 to March 2023, preoperative DTI and GQI examinations of 44 patients who underwent DBS surgery, were analyzed. To evaluate motor functions, the Unified Parkinson’s Disease Rating Scale (UPDRS) during on- and off-medication and Parkinson’s Disease Questionnaire-39 (PDQ-39) scales were used before and three months after DBS surgery. The study population was divided into two groups according to the improvement rate of scales: ≥ 50% and < 50%. Five target regions, reported to be affected in PD, were investigated. The parameters having statistically significant difference were subjected to a receiver operating characteristic (ROC) analysis.

**Results:**

Quantitative anisotropy (qa) values from globus pallidus externus, globus pallidus internus (qa_Gpi), and substantia nigra exhibited significant distributional difference between groups in terms of the improvement rate of UPDRS-3 scale during on-medication (p = 0.003, p = 0.0003, and p = 0.0008, respectively). In ROC analysis, the best parameter in predicting DBS response included qa_Gpi with a cut-off value of 0.01370 achieved an area under the ROC curve, accuracy, sensitivity, and specificity of 0.810, 73%, 62.5%, and 85%, respectively. Optimal cut-off values of ≥ 0.01864 and ≤ 0.01162 yielded a sensitivity and specificity of 100%, respectively.

**Conclusion:**

The imaging parameters acquired from GQI, particularly qa_Gpi, may have the ability to non-invasively predict the clinical outcome of DBS surgery.

## Introduction

Parkinson’s disease (PD) is the second most common progressive neurodegenerative disorder after Alzheimer's disease [21]. Around 1% of the population aged over 60 years is affected by PD, with its prevalence reaching approximately 6 millions in 2016 [12, 37]. Currently, it stands as the fastest-growing neurological disorder globally [11]. The primary histopathologic feature of PD involves the loss of dopaminergic neurons in the substantia nigra (SN) and the accumulation of Lewy bodies inside neurons, ultimately leading to neuroglial cell damage and demyelination of neuronal axons. Decreasing dopaminergic output in corticobasal and thalamocortical neuronal pathways causes dysregulation in motor functions [21]. Hereby, PD cases typically refer to clinics with motor symptoms like rigidity, refractory tremor, bradykinesia, and postural instability [2]. In addition to these, PD cases may also exhibit non-motor symptoms such as cognitive impairment, sleep behavioral problems, depression, and olfactory dysfunction [2]. As PD progresses to its late stage, individuals often experience significant limitations in their activities of daily living and become fully dependent on other people for assistance [29].

Deep brain stimulation (DBS) is a well-established, minimally invasive, and frequently utilized surgical intervention for PD. In this procedure, electrodes are implanted in the brain to administer electrical pulses, effectively suppressing abnormal activities and/or stimulating underactive pathways [23, 24]. The most frequently targeted brain region in DBS is the subthalamic nucleus (STN), which is an integral hub within the motor circuitry [10]. Since its initial implantation in the 1990s, DBS of the STN has been recognized as an effective treatment approach for PD [16]. By modulating neural pathways, DBS has been shown to make improvements in motor dysfunctions and, in certain cases, reduce the need for medication [19, 23, 24]. Clinical evaluation of the response to DBS surgery involves the use of various scales, such as the unified Parkinson's Disease Rating Scale (UPDRS) and Parkinson's Disease Questionnaire-39 (PDQ-39). The potential ability to predict the clinical outcome of DBS surgery before the implantation with non-invasive imaging techniques could significantly improve the clinical management, treatment strategies, and patient selection for DBS surgery.

Diffusion tensor imaging (DTI) and generalized q-sampling imaging (GQI) are two of the advanced magnetic resonance imaging (MRI) techniques that provide a non-invasive assessment of the microstructural integrity of the brain [36, 44]. Previous DTI studies in PD have demonstrated abnormal metrics in multiple brain regions, particularly in the dopaminergic pathways [45]. These studies generally focus on the role of DTI metrics obtained from various brain regions in the diagnosis of PD, primarily aimed at distinguishing PD from healthy control groups and Parkinson-plus syndromes [12, 16, 34, 45]. Another area of interest in the literature is the analysis of the correlation between DTI metrics and clinical symptomatology [7, 9, 14, 17, 20, 22, 26, 28, 39]. This study differs from previous research and aims to determine whether preoperative DTI and GQI parameters could non-invasively and accurately predict the postoperative clinical effect of DBS surgery prior to implantation in patients with PD.

## Material and methods

### Study design

This single-center retrospective study was conducted in accordance with the Declaration of Helsinki and approved by the local ethics committee of Basaksehir Cam and Sakura City Hospital (Protocol No: 2023–85; decision date: 22/02/2023). The requirement for written informed consent was waived by the ethics committee due to the retrospective design.

The inclusion criterion for this study comprised patients older than 18 years with a definitive diagnosis of clinically idiopathic PD with a history of DBS procedure. The following criteria determined exclusion: *i*, history of cranial surgery or chemoradiotherapy before MRI examination; *ii*, patients with a diagnosis of brain neoplasms; *iii*, interruption of the 3rd-month follow-up examination after DBS procedure; *iv,* lack of patient compliance for assessment of the 3rd-month follow-up examination; and *v*, missing radiological data.

**Fig. 1:**
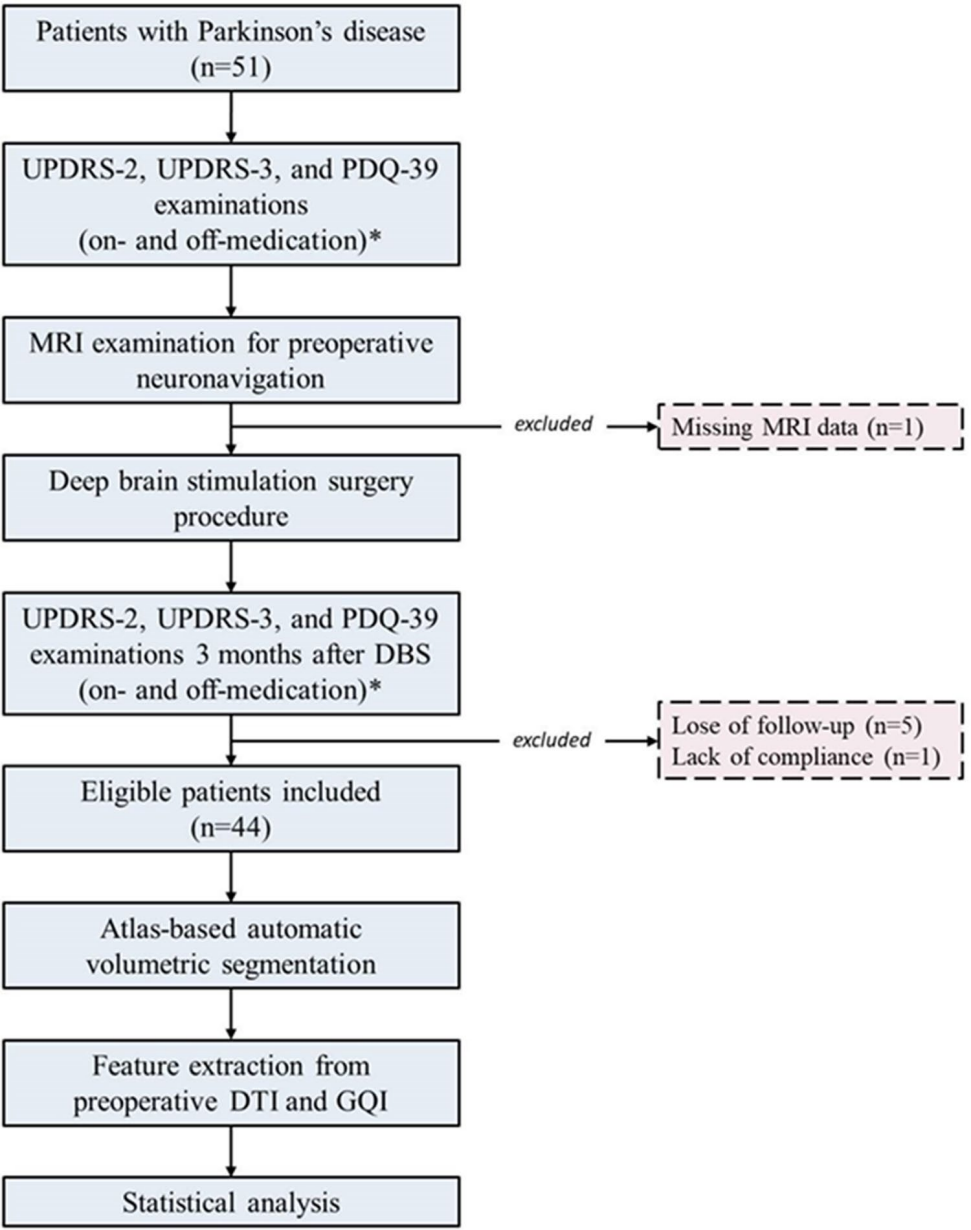
A summary flowchart of the study. *, only for UPDRS 2 and 3. *MRI*, Magnetic resonance imaging; *UPDRS*, Unified Parkinson’s Disease Rating Scale; *PDQ-39*, Parkinson’s Disease Questionnaire-39; *DBS*, deep brain stimulation; *DTI*, diffusion tensor imaging; *GQI*, generalized q-sampling imaging

Figure 1 presents the key aspects of the study design.

### MRI technique

All MRI examinations were performed 1–3 days before the DBS procedure, utilizing a 3.0 Tesla MRI unit (Ingenia, Philips, Best, The Netherlands) with 32-channel phased-array head coils in the supine position. MRI examinations were obtained for preoperative neuronavigation. MRI dataset were acquired in a parallel orientation to the anterior commissure-posterior commissure (AC-PC) line, extending from the vertex to the skull base. A routine MRI protocol consisted of DTI, T2-weighted imaging from 3D BrainVIEW sequence, and T1-weighted imaging from 3D Turbo Field Echo (TFE) sequence with the administration of gadolinium-based contrast agent.

Diffusion tensor imaging was acquired using a diffusion sequence (DTI_high_iso) with a b-value of 800 s/mm^2^, 32 diffusion sampling directions, and the following parameters: repetition time, 3945.52; time to echo, 93.70 ms; FOV, 22 cm; slice thickness, 2.5 mm; matrix, 110 × 112; acceleration factor, 2.5; in-plane resolution, 1.75 mm; with an acquisition time of 10 min and 50 s. The MRI sequence parameters are detailed in Table 1.

**Table 1:** MRI examination sequence parameters

Imaging Parameter	DTI	T2WI	T1WI
TR (ms)	3945.52	2500	8.09
TE (ms)	93.70	252.83	3.71
Flip angle	90	90°	8°
NEX	-	1	1
Matrix	110 × 112	252 × 252	252 × 240
Slice thickness (mm)	2.5	1	1
FOV (cm)	22 × 22	25 × 25	25 × 25

*DTI* diffusion tensor imaging, *T2WI* T2-weighted image, *T1WI* T1-weighted image, *TR* repetition time, *TE* time to echo, *NEX* number of excitation, *FOV* field of view

### DBS surgery procedure

One day before the operation, MR images were fused using Brainlab Elements Software (Brainlab, Munich, Germany). During planning, the STN was directly targeted with the assistance of automatic segmentation. A trajectory suitable for this target was calculated to complete the planning process. On the morning of the operation, patients underwent computed tomography (CT) imaging (Supria 128 Slice, Fujifilm Healthcare) with a 1 mm thickness after mounting either an Integra CRW Stereotactic Frame (Integra, New Jersey, USA) or a Leksell Frame (Elekta Inc., Stockholm, Sweden). Then, these CT images were fused with the previously obtained MR images in the planning station to obtain the stereotactic coordinates for targeting. The patient was prepared for the operation table under local anesthesia. Following adjusting according to the stereotactic frame coordinates, microelectrode recording and macrostimulation were performed on both sides sequentially. Finally, the lead was placed. Directional leads were used for all patients. On the same day, under general anesthesia, a pulse generator was implanted to conclude the operation. Six hours after the operation, a CT scan with a 1 mm thickness was conducted and fused with the planning images to verify the lead position and also to assess for any postoperative complications. If there was a deviation greater than 2 mm in the lead position, it was considered as lead misplacement.

### Image processing and analysis

All anonymized MRI data were analyzed by a neuroradiologist with six years of experience in the field of neuroradiological imaging. The radiologist was blinded to all clinical information of the patients during the analysis. DSI Studio software (Aug 30, 2021 build) was used to process diffusion imaging data using the DTI and GQI methods [43, 44]. The diffusion data were reconstructed using GQI with a diffusion sampling length ratio of 1.25. Then, GQI metrics were obtained, in addition to DTI metrics [43, 44]. The DTI metric used was fractional anisotropy (fa), while GQI metric was quantitative anisotropy (qa); representing the most commonly used metric for each technique [44].

### Segmentation

**Fig. 2:**
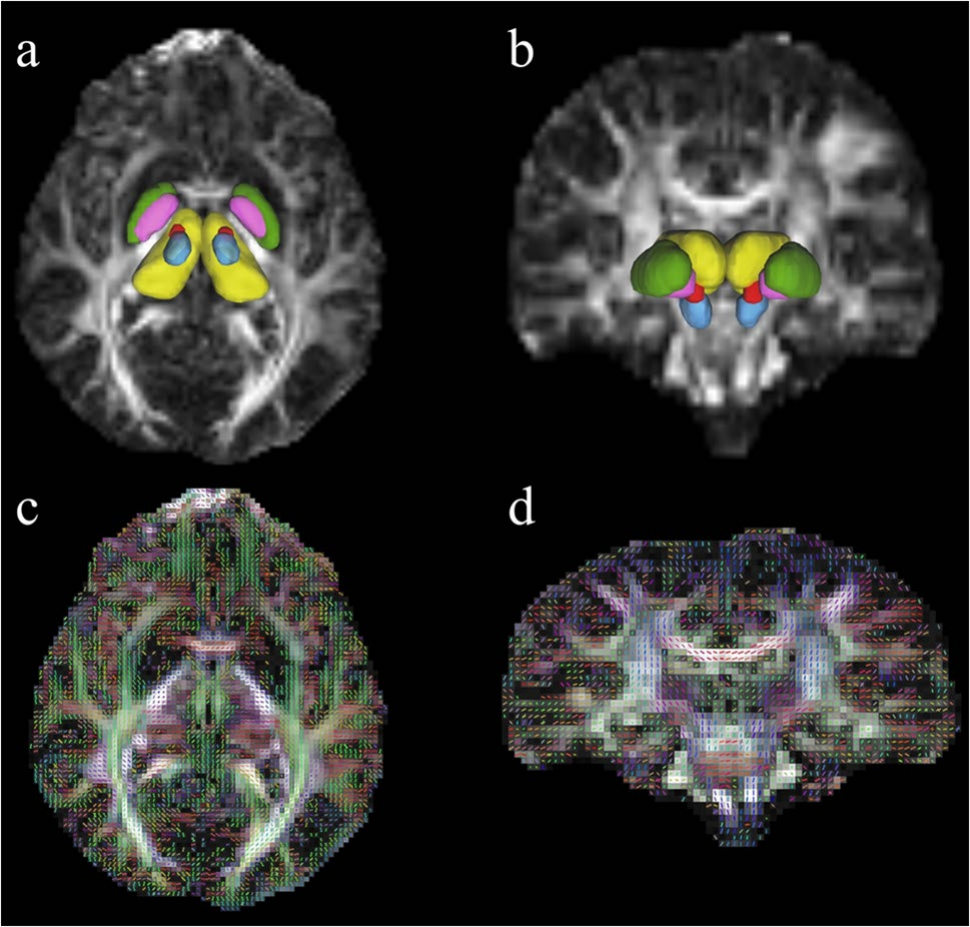
In generalized q-sampling imaging (GQI) examination, axial **(*a*)** and coronal **(*b*)** quantitative anisotropy (qa) map images illustrate the segmentation of the target regions: red for the subthalamic nucleus, yellow for the thalamus, green for the globus pallidus externus, purple for the globus pallidus internus, and blue for the substantia nigra. In the bottom row **(***c* and *d***)**, qa maps reconstructed by GQI, with fibers color-coded by direction

Firstly, both the subthalamic nucleus (STN), a crucial target in DBS, and subsequently, other brain regions including the thalamus, globus pallidus externus (Gpe), globus pallidus internus (Gpi), and substantia nigra (SN), which have been reported to be affected in PD, were volumetrically segmented [35]. All segmentations were conducted automatically, which is a notable strength of this study, using the atlas of ICBM-152 space [42]. Figure 2 shows the volumetric segmentation process.

### Imaging and clinical parameters

In total, 5 different regions were segmented bilaterally. For each target region, mean values of fa and qa were recorded. Hereby, a total of 10 imaging parameters (5 for DTI; 5 for GQI) were recorded. The patient's age, gender, and disease duration constituted three clinical parameters. As a result, a total of 13 parameters (10 imaging and 3 clinical) were recorded for analysis.

### Efficacy measures

Clinical examination of the patients was evaluated by experienced neurologist and neuropsychologist. UPDRS-2, UPDRS-3, and PDQ-39 scales of each case were recorded both preoperatively and at 3 months after the DBS surgery [40]. The evaluation of UPDRS-2 and UPDRS-3 scales were performed under two conditions: on-medication, when patients experienced the maximum clinical benefit after the usual dose of anti-Parkinson medication, and off-medication, when patients refrained from taking any medication for at least 12 h. In statistical analysis, the study population was divided into two groups in terms of improvement rates: 50% or higher and below 50%.

### UPDRS

The Unified Parkinson's Disease Rating Scale (UPDRS) is the most commonly used clinical scale in Parkinson's disease. It is widely accepted as the "Gold Standard" for recording and communicating the severity of a patient's symptoms [1]. The Movement Disorder Society (MDS) has modified the UPDRS grading scale (MDS-UPDRS) to be more comprehensive and consistent, with a focus on separating milder impairments and disabilities. To maintain thoroughly validated and clinically relevant scores, the MDS-UPDRS Task Force recommended presenting each part (Part#1–4) separately. The MDS-UPDRS includes ratings for 50 items.

Part#1 of the MDS-UPDRS focuses on "evaluation of non-motor experiences of daily living," comprising six semi-structured interview items and seven self-reported items. Part#2 deals with "self-evaluation of the motor activities of daily living," comprising 13 self-reported items with a score range of 0–52 (mild < 12, severe > 30). Part#3 involves "motor examination of parkinsonism signs" and includes 18 items with a total of 33 individual measurements, with a score range of 0–133 (mild < 32, severe > 59). Lastly, Part#4 addresses "motor complications" and consists of six semi-structured interview items [1, 13, 30]. To calculate the total scores for each MDS-UPDRS Part#1–4, the item scores are summed together. Each item is assigned a value between 0 and 4, with 4 indicating severe impairment and 0 indicating a normal score [15]. In this study, the reason for exclusively utilizing UPDRS 2 and 3 scores was to measure the impact of DBS surgery solely on motor functions and activities of daily living. Non-motor issues and side effects were not the primary focus of this study. Although there are different scales (e.g., Hoehn-Yahr and Schwab-England), we preferred to analyze UPDRS 2 and 3 scores, considering them the most suitable system for consistency with existing literature and clinical practice [1, 13, 30].

### PDQ-39

The Parkinson’s Disease Questionnaire (PDQ-39) is a self-reported assessment tool used to evaluate health-related quality of life in patients with PD. It consists of 39 items, categorized into 8 different domains. Respondents rate each item on a scale from 0 to 4, where 0 represents "never" and 4 indicates "always." To calculate the total score, the scores of all the questions are added together, divided by the highest possible score, and then multiplied by 100. Higher scores on the PDQ-39 indicate a significantly worsened health-related quality of life [15].

### Statistical analysis

The statistical analysis was conducted using Jamovi (version 2.2.5). Considering the normality of distribution, descriptive statistics are presented using mean, median, standard deviation, and interquartile range. *Shapiro Wilk test* was used to determine the normality of distribution. According to the number of cells in contingency tables, *Chi-square test* or *Fisher Exact test* was used to evaluate the difference between categorical variables. Depending on the distributions of the groups, a parametric or non-parametric test was used to assess statistical differences between groups in continuous variables. P values below 0.05 were regarded as statistically significant. To prevent potential false discoveries, *Bonferroni correction* was applied to multiple comparisons*.* Variables with statistical significance in univariate analysis were also evaluated using receiver operating characteristic (ROC) curve analysis. The optimal diagnostic performance cut-off values were determined using the *Youden’s index*. Besides, additional cut-off values were calculated to achieve maximum sensitivity and specificity (i.e., 100% for each).

## Results

### Baseline characteristics

Between September 2021 and March 2023, a cohort of 51 patients diagnosed with clinically idiopathic PD, who underwent DTI and GQI examinations before DBS surgery, were assessed for eligibility. Seven patients were excluded for the following reasons: interruption of the 3rd-month follow-up examination after DBS procedure (n = 5); lack of patient compliance for assessment of 3rd-month follow-up examination (n = 1); and missing MRI data (n = 1). In this study, a total of 44 patients were included. There was no significant difference between groups regarding age or disease duration (p > 0.05). There was no statistically significant distributional difference in sex for improvement rate of UPDRS-2, UPDRS-3, and PDQ-39 scales during on or off-medication (p > 0.05). Baseline characteristics related to demographic features and clinical scales for all patients are presented in Table 2 and Table 3, respectively. In this study, all patients' DBS leads were placed bilaterally in STN.

**Table 2:** Demographic features of all patients

Feature	Value	Range
Age (years), median (IQR)	59 (12.5)	35–70
Gender (male/female)	25/19	-
Disease duration (years), median (IQR)	10.5 (7)	4–35

*IQR* interquartile range

**Table 3:** Baseline characteristics of clinical scales for all patients

Clinical scale	Condition	Value
UPDRS-2 preoperative, mean (SD); median (IQR)	on-medication	9.1 (3.8); 10 (4.5)
	off-medication	25.5 (7.1); 26.5 (7.7)
UPDRS-2 postoperative, mean (SD); median (IQR)	on-medication	4.8 (3.5); 4.5 (4.2)
	off-medication	14.2 (7.3); 14 (8.2)
UPDRS-2 improvement rate (%), mean (SD); median (IQR)	on-medication	40 (47.3);50 (49.2)
	off-medication	41.4 (34.5); 42.4 (32.9)
UPDRS-3 preoperative, mean (SD); median (IQR)	on-medication	17.5 (10); 17 (14.2)
	off-medication	45.7 (14.3); 46 (19.5)
UPDRS-3 postoperative, mean (SD); median (IQR)	on-medication	8.1 (5.9); 6 (9.2)
	off-medication	20.7 (12.3); 17.5 (12.5)
UPDRS-3 improvement rate (%), mean (SD); median (IQR)	on-medication	34.7 (71.1); 59.7 (44.8)
	off-medication	51.4 (30.5); 59.6 (36.5)
PDQ-39, mean (SD); median (IQR)	preoperative	82 (24.7); 84.5 (21.2)
	postoperative	60 (28.6); 53 (42.5)
PDQ-39 improvement rate (%), mean (SD); median (IQR)	-	23.2 (40); 27.7 (38.2)

*UPDRS* Unified Parkinson's Disease Rating Scale, *PDQ-39* Parkinson's Disease Questionnaire-39, *SD* standard deviation, *IQR* interquartile range

### Univariate analysis of imaging and clinical features

For the analysis of group differences, a total of 13 parameters (5 DTI, 5 GQI, and 3 clinical) were evaluated. To assess statistical differences between groups, a corrected statistical significance threshold of 0.00385 (0.05/13) was applied according to *Bonferroni* method. Notably, statistically significant distributional differences were observed between the groups for three following GQI parameters in terms of the improvement rate of UPDRS-3 scale during on-medication: qa values from globus pallidus externus (qa_Gpe), qa values from globus pallidus internus (qa_Gpi), and qa values from substantia nigra (qa_SN) (p = 0.003, p = 0.0003, and p = 0.0008, respectively). For improvement rates of other clinical scales (i.e., UPDRS-2 during both on- and off-medication, and PDQ-39) and UPDRS-3 during off-medication, a statistically significant difference was not observed for the imaging and clinical parameters.

### ROC analysis

Parameters demonstrating statistical significance in group-based analysis were subjected to receiver operating characteristic (ROC) curve analysis. In the ROC analysis, the positive class was defined as an improvement rate of 50% or higher. The highest area under the ROC curve (AUC) was obtained from qa_Gpi with a value of 0.810 (AUC = 0.760 for qa_SN and AUC = 0.731 for qa_Gpe). Consequently, only qa_Gpi was further analyzed considering its highest AUC value.

**Fig. 3:**
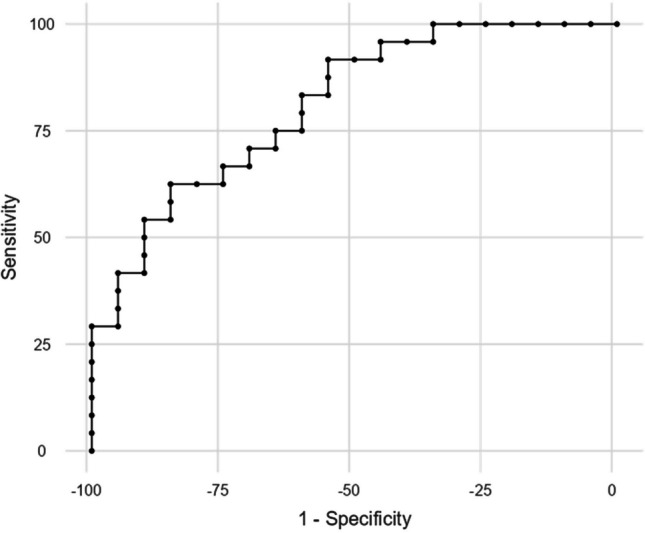
Receiver operating characteristic (ROC) analysis for qa_Gpi. The positive class was defined as an improvement rate of 50% or higher. *qa*, quantitative anisotropy; *Gpi*, globus pallidus internus

**Fig. 4:**
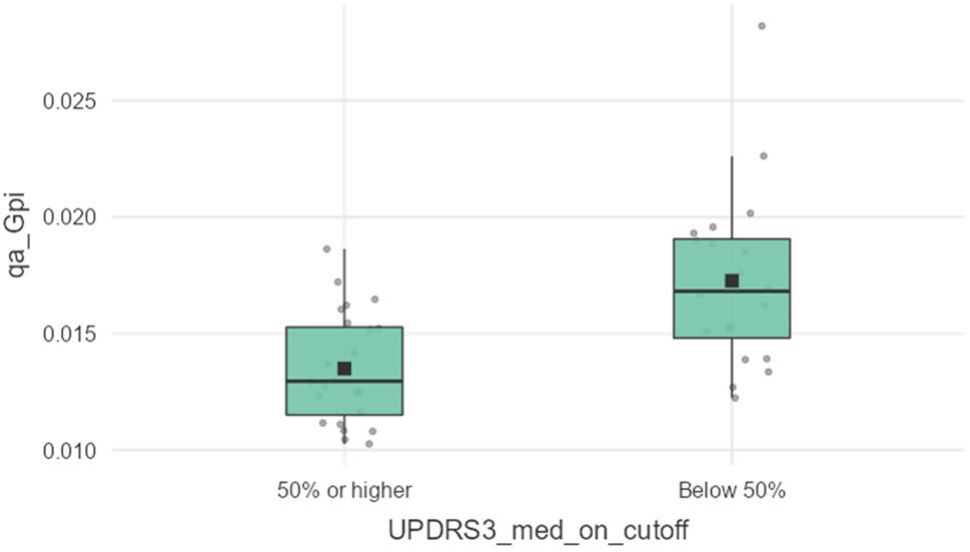
Box plots for qa_Gpi following univariate statistics. *qa*, quantitative anisotropy; *Gpi*, globus pallidus internus; *UPDRS*, Unified Parkinson’s Disease Rating Scale

Subsequent analysis revealed a sensitivity of 100% with a cut-off value ≥ 0.01864 and specificity of 100% with a cut-off value ≤ 0.01162. Maximizing the accuracy with *Youden’s index*, the chosen optimal cut-off value of 0.01370 achieved an AUC, accuracy, sensitivity, and specificity of 0.810, 73%, 62.5%, and 85%, respectively. Table 4 presents a comprehensive list of performance metrics for qa_Gpi with optimal cut-off values, while Fig. 3 shows the ROC curve for qa_Gpi. The confusion matrix for the optimal cut-off value, maximizing the *Youden’s index*, is presented in Table 5. Descriptive group statistics and box plots of qa_Gpi are presented in Table 6 and Fig. 4, respectively.

**Table 4:** Predictive performance of qa_Gpi for improvement rates of UPDRS-3 scale during on-medication according to optimal cut-off values

Strategy	Cut-off	AUC	Accuracy	Sensitivity	Specificity	Youden's index
Maximizing specificity	0.01025	0.810	48%	4.2%	100%	0.042
	0.01044	0.810	50%	8.3%	100%	0.083
	0.01079	0.810	52%	12.5%	100%	0.125
	0.01084	0.810	54%	16.7%	100%	0.167
	0.01110	0.810	57%	20.8%	100%	0.208
	0.01116	0.810	59%	25%	100%	0.250
	0.01162	0.810	61%	29.2%	100%	0.292
Optimal	**0.01370**	**0.810**	**73%**	**62.5%**	**85%**	0.475*
Maximizing sensitivity	0.01864	0.810	70%	100%	35%	0.35
	0.01885	0.810	68%	100%	30%	0.3
	0.01898	0.810	66%	100%	25%	0.25
	0.01931	0.810	64%	100%	20%	0.2
	0.01958	0.810	61%	100%	15%	0.15
	0.02016	0.810	59%	100%	10%	0.1
	0.02261	0.810	57%	100%	5%	0.05
	0.02821	0.810	54%	100%	0%	0

^*^ The optimal diagnostic performance cut-off value was determined using the maximizing Youden’s index

*qa* quantitative anisotropy, *Gpi* globus pallidus internus, *UPDRS* Unified Parkinson's Disease Rating Scale, *AUC* area under the receiver operating characteristic curve

**Table 5:** Confusion matrix of the optimal cut-off value maximizing the Youden’s index

		Predicted	
		Negative	Positive
Actual	Negative	17 (TN)	3 (FP)
	Positive	9 (FN)	15 (TP)

*TN* true negative, *FP* false positive, *FN* false negative, *TP* true positive

**Table 6:** Descriptives of the best imaging parameter with statistically significant difference. These results are for improvement rates of UPDRS-3 scale during on-medication. *UPDRS*, Unified Parkinson's Disease Rating Scale

Feature	Improvement rate	N	Median	IQR	Statistic	*p*
qa_Gpi	below 50%	20	0.017	0.004	91.000	0.0003
	50% or higher	24	0.013	0.004		

*qa* quantitative anisotropy, *Gpi* globus pallidus internus, *IQR* interquartile range

### Side effects and complications

In this study, no major surgical complications were encountered. Although some minor complications arose, they were effectively managed without the need for lead explantation. Stimulation-related side effects were noted in 11 (25%) cases: 5 cases exhibited a lateral capsular effect, which was rectified by adjusting the lead direction medially. Additionally, spontaneous resolution of dyskinesia occurred in 2 cases, while 2 cases presented with psychotic symptoms that regressed with medication. One case exhibited a lead location error exceeding 2 mm, promptly corrected two days later to ensure the placement within 2 mm of the intended target. Memory problems surfaced in one case at the 3rd month postoperative assessment, successfully addressed by reducing voltage and adjusting the lead direction posteriorly. Overall, surgical complications were observed in only 5 (11%) cases, encompassing non-recurrent epileptic seizure in 3 cases, spontaneously regressing peri-lead edema in 1 case, and asymptomatic caudate nucleus infarction in 1 case.

## Discussion

### Study overview

In the present study, we investigated whether DTI and GQI parameters can non-invasively predict the clinical outcome of DBS surgery in patients with Parkinson’s disease using various clinical scales. We found that the quantitative anisotropy values from globus pallidus internus (qa_Gpi) can non-invasively and correctly predict the improvement rate of UPDRS-3 scale during on-medication with an AUC, accuracy, sensitivity, and specificity of 0.810, 73%, 62.5%, and 85%, respectively. We also found that it is possible to achieve sensitivity and specificity of 100% by selecting specific cut-off values. Although we found distributional differences between groups for 3 different GQI parameters, we obtained the best predictive performance from quantitative anisotropy measurements obtained from the Gpi. The prominence of the Gpi in the study was not surprising. It is highly associated with motor symptoms and therefore with UPDRS-3 score [25]. Deep brain stimulation of Gpi has also been shown to be effective in improving motor symptoms. Moreover, it has been demonstrated that Gpi has a greater impact on motor fluctuations and motor experiences of daily living [25]. The Gpi results may be associated with the correlated role of both Gpi and STN in motor excitatory and inhibitory processes. These two nuclei may share the same pathways; for example, they are directly interconnected through pathways such as the ansa lenticularis and lenticular fasciculus [27, 31]. For these reasons, both STN-DBS and Gpi-DBS may represent another example of why they are beneficial in Parkinson's disease [31].

### About DTI and GQI

Diffusion tensor imaging (DTI) is an advanced MRI technique that allows the indirect assessment of microstructural integrity, orientation, and anatomical connectivity of the brain parenchyma in neurodegenerative diseases [3, 38]. The most commonly used quantitative metric of DTI to describe microstructural integrity is fractional anisotropy (fa). Fractional anisotropy, which measures the degree of diffusion anisotropy, is the most frequently used metric of DTI to assess the degree of axonal myelination, nerve fiber arrangements, and white matter tract integrity [6, 8]. Fractional anisotropy measures the level of water molecule anisotropy. When unimpeded, water molecules diffuse unhindered in all directions, but their trajectory can be influenced by barriers like macromolecules and cell membranes. Within an axon, diffusion only occurs along its axis, rendering it anisotropic rather than isotropic. By measuring this anisotropy, we can deduce changes in axonal integrity, density, or myelin structure [6, 8, 46]. To better characterize complex regions and separate fiber orientations, several new diffusion-based imaging techniques have been developed, providing an opportunity for more accurate descriptions compared to DTI [36]. One of these techniques is generalized q-sampling imaging (GQI), derived from q-space imaging, which offers more robust tensor metrics. The most commonly used metric in GQI is quantitative anisotropy (qa), associated with axonal density [41, 43, 46]. GQI can provide more precise and sophisticated details about diffusional changes by applying it to a broader range of q-space datasets, compared to DTI [43, 46]. While traditional DTI techniques may offer imprecise characterizations of local crossing and diverging fibers, the GQI method furnishes superior directional and quantitative insights through q-space datasets [46]. Studies have demonstrated that GQI can more effectively and clearly visualize nerve fibers in regions affected by cerebral edema, and it can depict nerve fibers in crossing regions more comprehensively and continuously compared to DTI [43, 46].

### Previous related studies

Numerous studies in the literature have focused on the diagnostic performance of DTI in PD. Many of these studies have centered on diagnosing PD and have demonstrated abnormal fa values in various brain regions, particularly within the dopaminergic nuclei and pathways, when compared to healthy controls [45]. Some studies have revealed a correlation between decreased fa values with neurodegeneration and degeneration due to dopamine loss [4, 12, 16, 33]. Some studies have also reported abnormal fa values in PD preceding brain atrophy, indicating the potential of DTI metrics as a biomarker for PD [18, 32]. A recent study conducted by Tsai et al. [34] investigated the diagnostic utility of the features extracted from DTI using machine learning. The findings revealed that these DTI-derived features exhibited a fair to very good diagnostic performance in distinguishing Parkinson's disease from healthy controls and Parkinson-plus syndromes.

In the literature, a limited number of studies have examined the correlations between DTI metrics and clinical symptomatology [14]. Some of these studies have reported significant associations between decreased fa values in the SN and increased severity of the motor symptoms, assessed through UPDRS-3 [6, 8, 33, 41, 46] or H&Y scale [7, 26]. Lenfeldt et al. [22] conducted a study involving 64 Parkinson's disease patients, analyzing DTI to explore alterations in diffusion metrics and their correlations with clinical rating scales. The results indicated that the thalamus and basal ganglia exhibited higher diffusion metrics with worsening clinical scales. UPDRS-2 showed a significant correlation with diffusion metrics, particularly mean diffusivity values from the globus pallidus and thalamus. However, UPDRS-3 scale did not demonstrate a significant correlation (p = 0.77), potentially influenced by medication effects.

Previous studies in the literature have not compared preoperative DTI metrics with improvement rate changes in motor scales following the DBS surgery, making it a unique and advantageous aspect of our study. Another distinctive feature of this research is the evaluation of three different scales (UPDRS-2, UPDRS-3, and PQS-39) and five different brain regions within the same study. Additionally, this study incorporates a metric derived from GQI, which is a novel and advanced diffusion-based MRI technique. To our knowledge, there is sparse radiological research literature on GQI and PD. Therefore, we aimed to share our knowledge to add new information about unpublished aspects of these two entities.

### Limitations

The present study has several limitations that need to be emphasized. First, it was a single-center and retrospective study, which may impact the generalizability of the findings. Second, medical dopaminergic treatments and the patient’s age may influence the clinical motor examinations, such as UPDRS, whereas these factors have less effects on DTI [45]. Third, clinical motor scales used in this study were not quantitative measurements and may depend on observers’ experiences. Fourth, various clinical conditions, including disease duration, disease severity, and physical therapy may potentially influence the clinical motor examinations. Fifth, the study's results were only based on the analysis of PD cases who had an improvement of > 30% of the UPDRS motor score with regard to dopaminergic medication before DBS surgery. It is a well-known inclusion criterion for DBS surgery to obtain optimal candidates [5]. Since it was a retrospective study, it was difficult to control all the conditions. However, it was possible to assess the correlation between on–off conditions in medication and stimulation. Sixth, this study only included patients treated with STN-DBS. Future studies may analyze similar predictive MRI parameters in Gpi-DBS patients, thus contributing to the determination of which of the two target locations (STN or Gpi) should be chosen for DBS surgery. Lastly, such a study could have been analyzed using logistic regression for binary outcomes, as in the current study, or even more specifically with linear mixed models for continuous improvement percentages. However, our small sample size prevented us from meeting the assumptions of these modeling techniques, so our analysis remained rather simple. We plan to continue the analysis with a larger cohort.

## Conclusion

In conclusion, the imaging parameters acquired from GQI may have the ability to non-invasively and correctly predict clinical outcomes of DBS procedures before surgery. Quantitative anisotropy values obtained from globus pallidus internus predicted the improvement rates of UPDRS-3 scale during on-medication with a good performance. Anticipating treatment response preoperatively may significantly enhance the patient selection for deep brain stimulation surgery. Further research with larger sample sizes is warranted to confirm our findings.

## Data Availability

The datasets generated during and/or analyzed during the current study are available from the corresponding author on reasonable request.

## Abbreviations

AUC: Area under the receiver operating characteristic curve
DBS: Deep brain stimulation
DTI: Diffusion tensor imaging
fa: Fractional anisotropy
GQI: Generalized q-sampling imaging
Gpi: Globus pallidus internus
MRI: Magnetic resonance imaging
PD: Parkinson’s disease
PDQ-39: Parkinson's Disease Questionnaire-39
qa: Quantitative anisotropy
ROC: receiver operating characteristic
SN: Substantia nigra
STN: Subthalamic nucleus
UPDRS: Unified Parkinson's Disease Rating Scale
